# T’ah kóó hóniidló, we’re still HERE! Mining legacies, Indigenous health and innovative solutions

**DOI:** 10.3389/fpubh.2024.1385325

**Published:** 2024-11-06

**Authors:** Ranalda L. Tsosie

**Affiliations:** Department of Earth & Environmental Science, New Mexico Institute of Mining and Technology, Socorro, NM, United States

**Keywords:** water filtration, Navajo Nation, water, health, mining legacies

## Abstract

In the world today, there are many unknowns especially with rising environmental concerns. However, one of the most important is an irreplaceable and shared resource, water or as the Diné (Navajo) refer to as, Tó. Throughout the world many Indigenous communities are facing water challenges, from lack of and access to adequate infrastructure, water rights, climate change and water contamination issues due to a variety of sources including anthropogenic sources like mining, especially, on the Navajo (Diné) reservation. This article aims to bring forth awareness of the long-standing water contamination issues in Diné communities and to shed light on innovative solutions being developed through current research efforts. Specifically, Dr. Tsosie’s research aims to optimize a handheld point of use filter unit with a filter casing design that is customizable to a community and/or household through an easily removable and exchangeable cartridge system. Despite all the challenges and legacies of mining faced by not only Diné communities but many Indigenous communities, We Remain and We Are Still Here.

## 1 Introduction

As a Diné scientist, I often reflect back on growing up on the Diné reservation and living without electricity and running water. This experience has given me a perspective that is much different than the average US citizen. I remember a time when my brothers and I would have to haul water from the local windmills for our livestock and our family’s everyday household needs. Unknowingly, we were drinking from contaminated water sources. Over the next few decades, I began to educate myself about the potential connections between contaminated water sources and their impact on human health after witnessing fellow community members succumb to a wide variety of health conditions with possible links to environmental contaminants. These events have given me the motivation to pursue remedies for ground and surface water contaminations in the form of point-of-use filters.

Since this study is aimed at addressing these contamination issues within a Diné community, it is important to practice proper protocols of Indigenous Research Methodologies (IRM). IRM is a conceptual framework, based on an Indigenous ontological foundation and epistemological approach (1–7). The approach is often developed by the researcher through recognizing their Indigenous worldview and approaching their research from a holistic, respectful and inclusive manner with the community in which they are conducting research. IRM begins by approaching the research at the community level first and understanding the perspectives and concerns of the community. In this study, IRM was applied by using Diné Worldviews to frame the scientific approaches and applying respectful collaborations with the community (8). The process of establishing respect and trust from the community is a priority. Developing these relationships was accomplished by hosting listening sessions and receiving community input. The protocols of gaining the respect and approval of the community to conduct the research on water sources was vital to the collaboration. Many of the concerns that arose from the community listening sessions were about human health impacts and impacts on livestock.

## 2 Background

### 2.1 History of the Diné and mining

The Navajo reservation (Diné Bikéyah) is located is the U.S. southwest and occupies the states of Arizona, New Mexico and Utah. It spans 27,000 square miles of “unparalleled beauty” (9). The population has grown to 399,494 enrolled tribal members with more than 166,000 that live on the reservation (10). The vast majority of the Navajo reservation is located on the Colorado Plateau (11). Considering the multiple campaigns by the U.S. government and military to remove the Diné from their homelands, we remain within our four sacred mountains that we hold reverent, a direct result of the resilient mindsets of our ancestors. The Diné did not go quietly and resisted removal for many years. The U.S. Army used tactics commonly labeled as “scorched earth policies” that destroyed more than 4,000 peach trees and 11,000 acres of both corn and beans (3).

The most prevalent and devastating issues, are the impacts and legacies from mining. The discovery of abundant natural resources on Diné lands, created an influx of prospectors and mining operations extracting coal, vanadium, uranium (U), copper, sand and gravel (12–22, 68, 69). These mining operations employed many Diné citizens who were not informed of the potential health hazards (12, 13). As these operations closed down, some were abandoned without proper cleanup. According to the Navajo Abandoned Mines Lands department, there are currently 273 coal sites, 33 copper mines and over 1,000 non-coal (uranium) abandoned mines, some have been and are being reclaimed (23). To date, several efforts are actively working with clean-up, education, and outreach. These efforts are difficult to address, expensive and require collaboration from local communities, tribal, state and federal agencies.

### 2.2 Metal impacts on human health

Numerous health effects that arise from exposure to heavy metals and metalloids including arsenic, uranium, lead, mercury, manganese, and others (12, 16, 18–22, 24, 25). These complex mixtures naturally occur in the environment and individuals are commonly exposed to these metals by inhalation, ingestion and in some cases through dermal contact. Comprehensive public health studies on the Navajo Nation report preliminary results that indicate chronic kidney disease, diabetes, high blood pressure and autoimmune disease are higher in Navajo communities with a higher number of uranium mines (26, 27). Initial exposure models indicate that environmental exposures, including living within 0.8 kilometer of a uranium mine site and coming in contact with wastes are significant predictors of kidney disease and diabetes (26, 27).

Uranium exposure has been linked to increases in cancer mortalities and has been demonstrated to have radiological effects in various organs such as bone, kidney, brain, liver, lung, intestine and reproductive systems (28–32). These effects are mainly due to uranium’s radiological (alpha emitter) and chemical properties. Thus, uranium toxicity results from both chemical and radiological toxicity (33). A link has been established between birth defects and adverse pregnancy outcomes for women living in close proximity of abandoned mines (30, 34–36). Some of these exposures occur from occupational exposure and through exposure to mine tailings. Some studies have also indicated that acute exposure to uranium can be chemically toxic to the kidney and chronic exposure can be genotoxic (37).

Similarly, arsenic is also an element of concern throughout the world due to its toxicity. According to the World Health Organization, arsenic persists in groundwater and natural water sources and is highly toxic in its inorganic form (38). The common form of exposure is through ingestion and can lead to numerous adverse health effects in humans such as neuropathy, developmental disabilities, numerous skin disorders, hypertension, various cancers (skin, lungs, bladder and kidney), cardiovascular disease and diabetes (38–42). Some studies suggest that there are synergistic effects through co-contaminates such as arsenic and selenium that naturally occur in the environment and are associated with other types of health problems and cancers (30, 38, 41, 43).

While both uranium and arsenic have established maximum contaminate limits (MCL) in drinking water, vanadium currently does not have any set standards in the U.S. Carcinogenicity of vanadium has not been identified in drinking water standards, health advisories list, nor health hazards lists. The exception is vanadium pentoxide which has an inhalation risk and standards that vary from 0.02 to 0.1 mg/m^3^, depending on the organization OSHA, NIOSH and the American Conference of Governmental Industrial Hygienists (44). In 2009, the U.S. EPA assessed the carcinogenicity of vanadium and concluded that an MCL cannot be specified. This is because there are no human data and inadequate animal studies with links to increased cancer mortalities and the potential carcinogenicity of soluble inorganic vanadium compounds (44, 60). However, chronic exposure to vanadium compounds can affect the respiratory system and causes irritation and more serious effects like bronchitis and pneumonitis (45). Determining exactly how vanadium contributes to respiratory related illnesses remains for the most part unknown. This area of vanadium research requires more *in vitro* and *in vivo* studies to determine the mechanisms of how it relates to respiratory illnesses. Additional studies are required to determine the overall effect of vanadium on both the environment and human health. Since vanadium concentrations are highest in soil, background levels similar to arsenic and uranium are created. Therefore, it is difficult to pinpoint the origin of the contamination in the environment.

### 2.3 Metal impacts on the environment

Water, a precious resource, is globally impacted because of anthropogenic sources like mining. While the geographic area of the Navajo Reservation has a natural abundance of metal and metalloids like uranium, vanadium and arsenic, the disturbance of these ores by mining has mobilized these elements, increasing levels in ground and surface waters. Numerous studies by US EPA, DOE, USGS and academic institutions have reported the extent of uranium contamination (8, 14–23, 31, 34–36, 39, 40, 42–59). Arsenic and vanadium are rarely mentioned in these studies although they pose an additional threat to human health. Anthropogenic activities do contribute to sources of metal contamination in water, soil and air (32). Aside from the greatest concern for water source contamination, the extent of soil and vegetation contamination is understudied. There is a growing concern for soil and vegetation contamination because of the potential implication that it can have harmful effects on livestock, traditional plants and medicines. If a soil is contaminated with uranium, arsenic and vanadium, then there is an increased possibility that the vegetative cover is also contaminated by uptake into the roots and other parts of the plant. El Hayek *et.al* demonstrated the uptake of uranyl in roots of *Brassica juncea* (46). Other studies have shown uptake of arsenic in chokecherries and other indigenous plants (70, 71). Currently, a series of studies are being conducted within Diné communities focusing on livestock, vegetation and agricultural products and the uptake of contaminating metals, so that these mechanisms are better understood, and a plan can be developed to address these community and tribal concerns. At the top of the list are concerns for the Diné livelihoods of ranching and agricultural economies.

### 2.4 Historical and current efforts to address mining legacies

In the last 30 years, there have been numerous efforts to understand the extent of contamination in soil and water within the boundaries of the Navajo Nation. To date, some of the most concerning sites have been reclaimed to a certain extent (23, 39, 40). The remediation efforts are on-going by tribal and federal agencies. Additionally, researchers like myself, are conducting studies to further understand the extent of contamination and attempting to provide Diné communities with answers (8, 47–49, 58, 59). Some studies are investigating livestock and wildlife (50–52). Others are exploring innovative approaches to temporarily address the water contamination (53–56, 61–63).

In 1994 and through 2000, the USEPA Region 9, U.S. Army Corp of Engineers (USACOE), Navajo Nation EPA Superfund Program (NNEPA), Bechtel Environmental and the U.S. Department of Energy (USDOE) Remote Sensing Laboratory investigated issues related to abandoned mines and the exposure to radiation and toxic metals that led to the Abandoned Uranium Mines Project. The overall objective of the investigation was to determine whether abandoned uranium and vanadium mines or related mine features posed a significant risk to human health and to identify areas or features requiring action to reduce exposure. It was determined that there were multiple pathways of exposure included water, soil, groundwater and combined pathways (57).

In 2013, Dias da Cunha *et.al* conducted studies on radionuclides in four small communities. They presented results that identified 19% of the water sources tested had uranium concentrations that exceeded MCL for total uranium (47). Furthermore, 14% of the water sources tested exceeded MCL for U^238^ and 17% for U^234^ (47). The Ingram group at Northern Arizona University have been involved in a number of studies that identified water sources exceeding the established MCL for both uranium and arsenic (8, 48–50). Out of New Mexico, the University of New Mexico Center for Native Environmental Health Equity groups has a long history of studying impacts of uranium, mining on human health, as well as a number of other environmental impacts (53). There are numerous research groups conducting studies on the impacts of mining on the Diné people.

One interesting area of study is the development of innovative solutions to address the water quality issues that these communities are facing. A collaboration between Navajo Technical University and New Mexico Institute of Mining and Technology, the Navajo Nation Water Purification Project (N^4^WPP), is currently developing desalinization units to provide an alternative and temporary solution for contaminated water sources (54). Researchers at the University of Arizona are also developing a similar technology, a solar powered desalinization unit that implements hollow fiber membranes to treat brackish waters (55). The University of Austin is collaborating with Diné citizens to impregnate silver nanoparticles with pine resin, used in traditional Diné pottery, as a means to remove bacteria from water (56). Collaborative research through the Center for Native Environmental Health Equity and Oklahoma State University is currently investigating the occurrence of microplastics and other products in open dump sites (53).

## 3 Development of innovative filtration technologies

Similarly, the Tsosie research group is developing a handheld water filtration unit for use by community water haulers. This project began as a vision and involved multiple partners from tribal communities to students. Tribal communities involved in this project, were involved by bringing forth concerns for their water sources. Past mining efforts to geologic conditions created challenging water insecurities and contamination issues that these communities continue to deal with. The point of use (POU) filter is designed with the user in mind. The filter is a handheld unit that is meant to travel with the user to unregulated water sources located, at times, miles apart. In the spring the availability of water is more widespread but as late fall approaches, the water becomes sparce. The POU filter will purify the water collected at these unregulated water sources, such as windmill wells. The filter ensures safe drinking water for livestock, for agricultural and household purposes. However, it is currently advised that these unregulated water sources should not be used for human consumption because these water sources are not regulated by the tribe.

In 2022, Dr. Tsosie collaborated with five mechanical engineering students, two students from civil engineering at Montana State University to design, develop and produce the handheld unit. An additional unit design feature is the customizable cartridge system. The unit has removable cartridges that allows for different solid phase extraction (SPE) materials to be changed and customized to the site. For example, the system could be used on the Navajo Nation, where the waters are high in arsenic, uranium and vanadium, and in Montana where the biggest challenge is nitrates and biological contaminates. The resin types can range from silica polyamine composites (SPCs) (8, 64–67), ion exchange resins, and other SPE materials that are currently on the market. SPCs are ideal since they are materials that can be regenerated. Thinking beyond just the Diné nation, the possibility of this filter being used to restore drinking water in communities facing similar contamination issues is something to aspire to for the well-being of future generations. This SPC-POU filter could have potential applications for elements of concern beyond just arsenic, vanadium and uranium. Examples include microplastics and per-and polyfluoroalkyl substances. This project is working on bench top studies to identify any unresolved issues with the unit. This could include issues with flow, increased pressure drops across the system, the potential need for a solar powered pump, and an indicator that identifies SPE materials are nearing saturation. In addition, studies are aimed at identifying additional SPE materials for selective removal of elements of concern.

These innovative technologies, led by Diné citizens, are addressing water challenges on the Navajo Nation and thereby addressing the health and environmental disparities being experienced by fellow tribal members. While the impacts and legacies from mining are great, there are also great minds attempting to resolve these issues. The process may be slow but progress is being made.

## 4 Conclusion

Throughout history Indigenous peoples have experienced so many hardships and setbacks, but we remain and we are still here. Through raising our voices and sharing our stories, we are able to bring change to our communities. Indigenous and non-Indigenous researchers alike are making a difference in our communities. Through efforts to bring awareness, promoting health and by creating innovative solutions, these communities are able to lessen the disparity gap. The examples brought forth in this perspective are a small portion of issues that are impacting Indigenous communities. The realm of environmental justice pertaining to Indigenous communities spans multiple areas, including water, soil, air, plants, animals, Indigenous data sovereignty, law, health, tribal rights and much more.

Incorporating IRM is vital to working *with* and *in* Indigenous communities. As the field of Indigenous scholars that are incorporating IRM into their research and practices is growing, it also becomes imperative that Indigenous Knowledge’s and worldviews are equitably included in academic spaces. There is an opportunity for Indigenous researchers like myself to demonstrate that Indigenous and western science/knowledge’s can cooperatively resolve problems in communities impacted by environmental injustices.

The intent and main motivation of the POU filter project was first and foremost the quality of life of the Diné people especially those living in close proximity of abandoned mines. It has been 38 years since the operations of these mines ceased and yet the Diné people continue to thrive despite the increased cancer mortalities, respiratory illness, diabetes and other diseases linked to the lingering effects of mining. The examples of new innovative solutions are a clear way forward. Finally, it does not matter who is at fault, but the efforts leading the charge to create change is more important.

WE ARE A RESILIENT & PROUD PEOPLE!

## Data Availability

The original contributions presented in the study are included in the article/supplementary material, further inquiries can be directed to the corresponding author.
